# Health amongst former rugby union players: A cross-sectional study of morbidity and health-related quality of life

**DOI:** 10.1038/s41598-017-12130-y

**Published:** 2017-09-28

**Authors:** Madeleine A. M. Davies, Andrew D. Judge, Antonella Delmestri, Simon P.T. Kemp, Keith A. Stokes, Nigel K. Arden, Julia L. Newton

**Affiliations:** 1 Arthritis Research UK Centre for Sport, Exercise and Osteoarthritis, University of Oxford, Nuffield Department of Orthopaedics, Rheumatology and Musculoskeletal Science, Oxford, UK; 2 Rugby Football Union, Twickenham, UK; 3 Arthritis Research UK Centre for Sport, Exercise and Osteoarthritis, Department of Health, University of Bath, Bath, UK

## Abstract

In the general population, physical activity is associated with improved health outcomes. However, long-term sports participation may be associated with adverse outcomes, particularly at the elite level. The aims of this study were to assess morbidity and health-related quality of life (HrQoL) amongst former rugby players, compared to an age-standardised general population sample. A cross-sectional study of former elite, male, rugby players (n = 259) was undertaken, and standardised morbidity ratios (SMR) calculated, assessing morbidity prevalence relative to English Longitudinal Study of Aging participants (ELSA, n = 5186). HrQoL, measured using the EQ-5D, was compared to a Health Survey for England (HSE, n = 2981) sample. In SMR analyses of participants aged 50+, diabetes was significantly lower amongst former players, (0.28, 95% CI 0.11–0.66), whereas osteoarthritis (4.00, 95% CI 3.32–4.81), joint replacement (6.02, 95% CI 4.66–7.77), osteoporosis (2.69, 95% CI 1.35–5.38), and anxiety (2.00, 95% CI 1.11–3.61) were significantly higher. More problems in HrQoL were reported amongst former players within the domains of mobility (p < 0.001), self-care (p = 0.041), usual activities (p < 0.001) and pain/discomfort (p < 0.001). Morbidity and HrQoL differ between players and the general population, with higher musculoskeletal morbidity and lower diabetes amongst former players. The magnitude of musculoskeletal morbidity may warrant proactive osteoarthritis management within this population.

## Introduction

Despite the known benefits of exercise and physical activity^1^, there is an increasing focus on the potential for contact sports such as rugby union to be detrimental to physical and neurological health and wellbeing^2–8^. Based on figures for global participation of over 7 million men, women and children in rugby union across more than 120 countries^9^, potential alterations in health status and health-related quality of life following participation could represent a significant public health benefit or burden.

Little is currently known about morbidity, healthy aging or health related quality of life amongst former rugby union (‘rugby’) players^2–4,10^. Injury incidence within rugby is higher than for non-contact sports, and differs between levels of play, with injury most common at the elite levels of play^11^. A recent meta-analysis suggested that injury incidence (>8 days timeloss) during match-play is higher in senior rugby union (81 injuries/1000 hours, 95% CI, 63–105)^12^, when compared to semi-professional, (22/1000 hours (95% CI, 20–24), amateur (17/1000 hours, 95% CI, 15–18) and recreational levels of play (14/1000 hours, 95% CI, 13–16)^13^. Comparatively, the injury incidence within popular non-contact participation sports of elite football and cricket has been reported as 8.0 and 2.7 injuries per 1000 match hours, respectively^14,15^. The impact of this higher injury incidence in rugby union on longer-term morbidity and health-related quality of life has not been established to date.

There is limited data on long-term musculoskeletal, neurological and general health outcomes following any sports participation, at any level of play^5,8,16–19^. Informed decision-making for current and future sports participation requires potential health benefits and adverse outcomes to be considered with a balanced approach, and presented together. This also provides sports governing bodies with a greater body of evidence to support their management of player safety. Decreased morbidity following sports participation can promote sport as a means of improving health. Where sport is associated with increased morbidity, identifying modifiable risk factors is fundamental to facilitating appropriate intervention, and increasing healthy sports participation for the highest number of participants. Given the effect of the level of play on injury rates, and more regulated and closely monitored nature of elite sport, health status may be most feasibly influenced at elite levels of sports participation.

The aims of this study were to establish: (1) the prevalence of morbidity and measure health-related quality of life within a cohort of former elite, male, rugby players, and (2) to quantify differences in morbidity between this sporting population and a representative age-standardised (50years+) general population comparator group.

## Methodology

### Ethical approval

The study received ethical approval by the University of Oxford Central University Research Ethics Committee (MSD-IDREC-C1-2014-020) and was conducted in accordance with the ethical standards of the Declaration of Helsinki, with informed consent obtained from all participants or their guardians.

### Patient and Public Involvement

Patient and public involvement was undertaken throughout the research cycle of this study, with rugby players assisting as consultants co-developing and managing this study^20^. Current and former players were extensively involved throughout the research cycle, with study and questionnaire design, study oversight, and questionnaire testing. This process aimed to ensure the questionnaire was accessible and intuitive for players, and that it was revised and tested with the target population. Two player involvement fora were undertaken with eleven players informing the recruitment strategy, research agenda and functionality of online data collection, the process and output of which have been reported elsewhere^20^.

### Study design

A cross-sectional study design was used to assess the prevalence of self-reported physician-diagnosed morbidity and health-related quality of life (EQ-5D), within former elite, male, rugby players. The prevalence of physician-diagnosed morbidity (asthma, diabetes, high blood pressure, heart problems, stroke, anxiety, depression, dementia, osteoporosis, osteoarthritis, joint replacement and hip and knee replacement), and reported problems with health-related quality of life (EQ-5D)^21^, were compared between former players and participants in nationally representative population-based studies. Elite players were selected due to their well established and preserved alumni playing records, which allowed us to determine the total number of potential participants available, and therefore accurate estimates of prevalence. They also represent those at the highest risk of injury^12^, who may have more regulated training, and higher playing exposures, during their career. Any health detriments attributable to rugby may therefore be most evident amongst this former elite playing population.

### Participants

#### Elite rugby cohorts

Eligible participants included former Oxford and Cambridge University players, as well as former English international players. Electronic recruitment of these cohorts through mailing lists was staged, and took place between August 2014 and February 2016. UK-based former Oxford players, with club-held postal addresses, were also sent a postal questionnaire. Former England player recruitment was through a membership organisation, the England Rugby International’s Club (ERIC), of which the membership includes approximately 90% of all living, former England International players. ERIC members without email addresses were contacted via post where these details were available. Participants were offered the opportunity to participate by postal questionnaire or telephone interview if preferred, and completion by proxy was permitted where requested.

**Figure 1:**
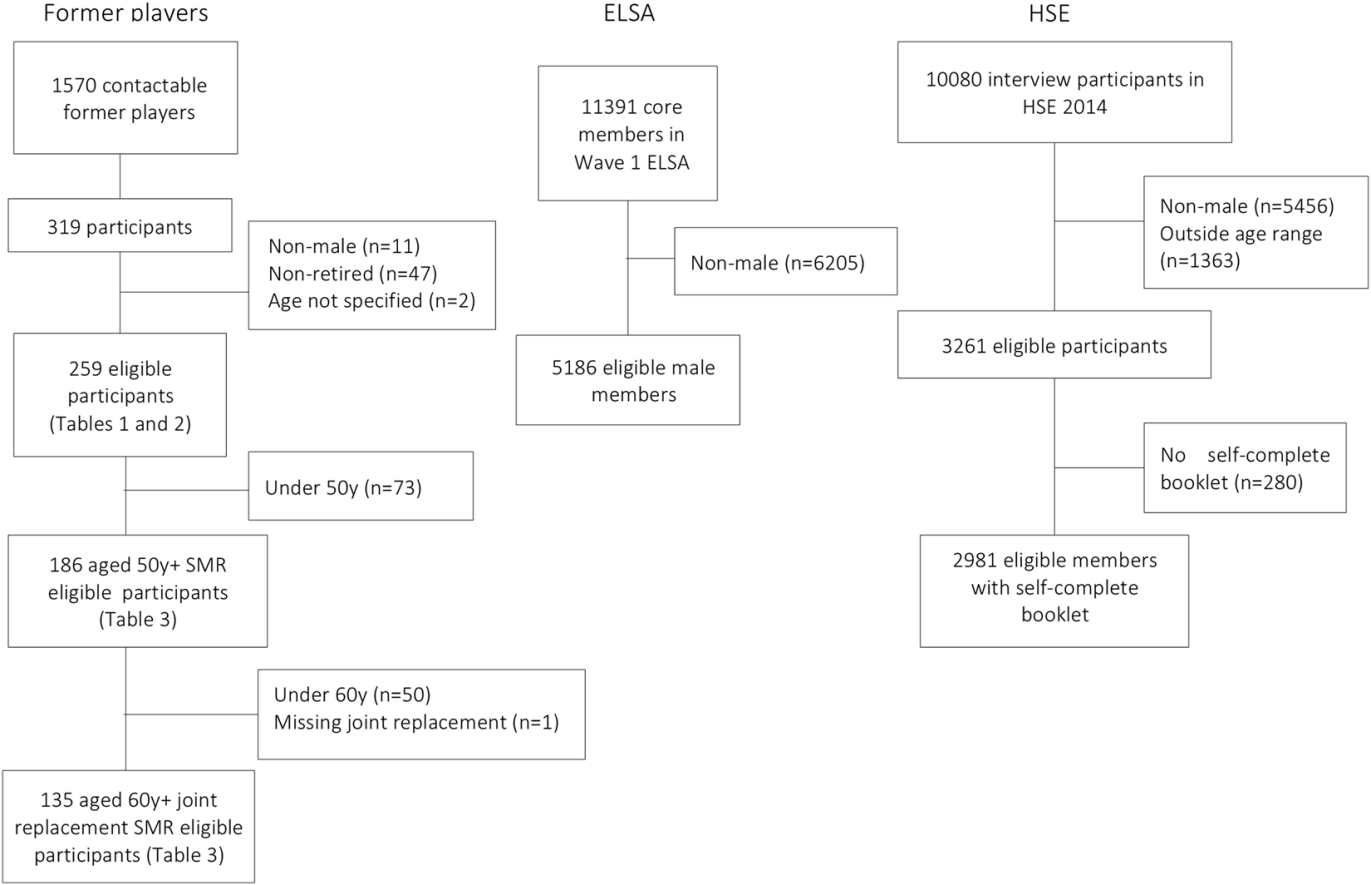
Participant flow from contactable players and comparison population participants to eligibility for this study and matched analyses.

Rugby participants not specifying male sex, retired playing status, or missing age (Fig. 1) were excluded from analyses. As rugby became a professional sport in relatively recently, amateur or professional playing status was assessed in order to ascertain the number of elite yet amateur participants within the sample, compared with those who had played predominantly during the professional era. Study data were collected and managed using a secure web application for Research Electronic Data Capture (REDCap)^22^, hosted at the University of Oxford.

#### General population cohort

Two datasets were used as representative population-based comparison cohorts, the Health Survey for England (HSE) and the English Longitudinal Study of Aging (ELSA). The HSE is an annual cross-sectional survey of adults and children living in England, while the ELSA is a longitudinal study which contacted HSE respondents from specific years (1998, 1999 and 2001), to participate in a prospective study of the health, social, economic circumstances and wellbeing of the English population, aged 50 years and older^23^. Specifically, the 2014 dataset of HSE was used as a cross-sectional, recent representative population-based survey comparator group for health-related quality of life (EQ-5D)^24^, whereas the first point of ELSA data collection (Wave 1), was used as a cross-sectional population-based comparator group, but for multiple morbidities (asthma, diabetes, high blood pressure, heart problems, stroke, anxiety, depression, dementia, osteoporosis, osteoarthritis, joint replacement and hip and knee replacement). Data from female participants were removed from both comparison datasets.

### Data sources/measurement

Participant age was categorised into ten-year age groups (20–29, 30–39, to 90–99) across all cohorts, and HSE participants outside of the age range of rugby participants (<24 years) were excluded (n = 1363), in addition to those who has not fully or partially completed the self-completion questionnaire booklet containing the EQ-5D (Fig. 1). It is important to note that age within HSE is capped such that all participants over 90 years of age are coded as 90 years.

The main outcome variables of self-reported, physician-diagnosed asthma, diabetes, high blood pressure, heart problems, stroke, anxiety, depression, dementia, osteoporosis, osteoarthritis, and joint replacement, hip and knee replacement were collected in rugby cohorts using a custom-designed questionnaire (Supplementary Table S1), and the prevalence of each morbidity estimated Variables were a priori selected as being contributors to poor health, and derived as binary variables. Observations which had a ‘don’t know’ response were recoded as missing at the disease level, and are indicated in the results section.

Within ELSA, reporting of cardiovascular-related or chronic diseases was assessed with one overarching question, extended to up to ten variables if conditions were applicable (see Supplementary Tables S1 and S2). To derive a parallel binary heart problems variable in ELSA, we combined the outcomes of angina, heart attack, heart murmur and abnormal heart rhythm. The phrasing of a specific morbidity had some variability between groups, such as ‘diabetes’ for rugby participants and ‘diabetes or high blood sugar’ in ELSA and ‘high blood pressure’ in rugby and ‘high blood pressure or hypertension’ in ELSA. Consensus on the appropriateness of a comparison given any variability in phraseology was discussed and agreed within the research time.

Certain variables collected in HSE (height, weight, BMI) were not collected at Wave 1 of ELSA, and were included in analyses after being extracted from Wave 0 of ELSA (participants’ prior HSE questionnaire). Data for Wave 0 and Wave 1 were made available through the UK Data Archive.

The EQ-5D was selected as a valid and reliable measurement of health-related quality of life, which has been seen to maintain validity and reliability across different geographical and disease populations^25–29^. The 5-level EQ-5D-5L was used for rugby participants due to its increased capacity to discriminate participants with slight, moderate or severe problems within a domain (mobility, self-care, usual activities, pain/discomfort and anxiety/depression). The EQ-5D-5L has also been shown to provide stronger evidence of validity compared with the EQ-5D-3L^30^. A nationally representative 5-level population comparison was not available at the time of analysis, and therefore the 2014 HSE, which used a 3-level EQ-5D-3L, was selected as a reference population.

Player sentiment was assessed with novel questions in the custom-designed questionnaire. These questions were devised to examine how players reflected on their playing career, and to understand if given the perceived benefits and risks of their playing exposure and experiences, whether players felt that they would undertake the same participation again, or recommend this to their friends or relatives.

### Statistical methods

Statistical analyses were undertaken using Stata 13.1. Descriptive statistics (mean (standard deviation) for continuous variables, and number (percentage) for categorical) are presented for the former rugby-playing population and the general population comparison groups. Between-group differences for ELSA and rugby, and HSE and rugby participants, respectively were tested using unpaired t-tests for continuous demographics, and chi-squared tests for categorical variables.

**Figure 2:**
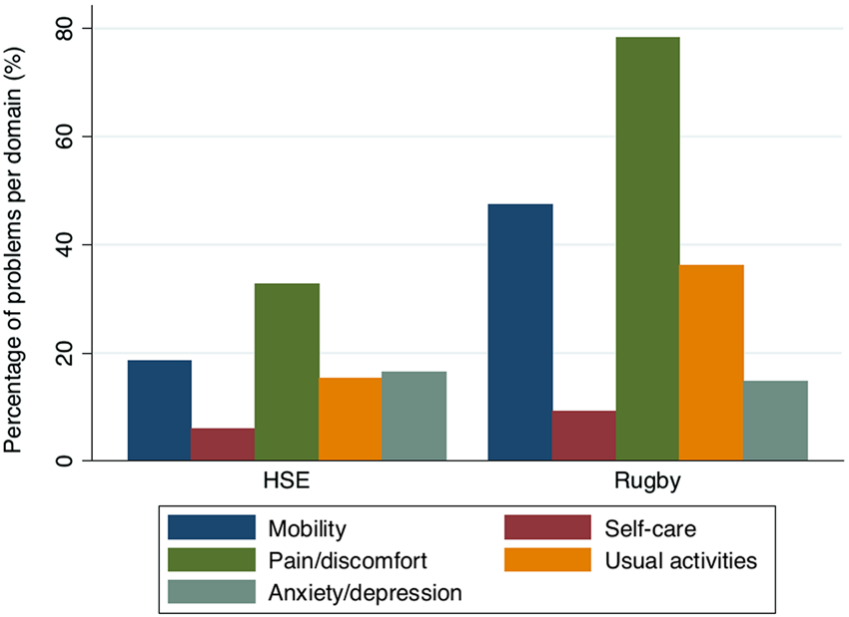
Percentage EQ-5D problems per dimension amongst HSE and rugby participants.

A priori analyses were the estimation of morbidity prevalence, age standardised morbidity ratios^31^, and descriptive statistics for reported problems within each domain of health-related quality of life. The prevalence of morbidity in former players (any age), players aged 50+, and ELSA participants was estimated, and then standardised morbidity ratios were used to demonstrate where morbidity prevalence amongst former players (aged 50 years+ or 60 years+ for joint replacement), exceeded or was inferior to that of the ELSA general population control group. Due to the different EQ-5D tools used (EQ-5D-5L and EQ-5D-3L), responses were dichotomised to a ‘no problems’ (EQ-5D ‘1’ scoring) or ‘problems’ (EQ-5D ‘2+’ scoring) variable, for each of the 5 dimensions (Fig. 2)^21^.

Post-hoc sensitivity analyses were included to utilise all player data, by examining significant differences in the prevalence of morbidity between the entire cohort of former, male and age-specified players of any age (n = 259), and ELSA participants (aged 50+), and testing for significant differences in morbidity between groups using logistic regression. Post-hoc chi-squared tests were used to determine if differences in the reported problems between players and HSE participants were statistically significant for each dimension of the EQ-5D.

### Data availability

The ELSA and HSE datasets that support the findings of this study are freely available but were used under license for the current study. Rugby data generated and analysed during the current study are not publicly available due to ethical limitations.

### Ethical approval

Ethical approval was granted by the University of Oxford Central University Research Ethics Committee (Ref. MSD-IDREC-C1-2014-020). Informed consent was provided by all study participants.

## Results

### Former players

From 1570 contactable players, 319 former Oxbridge and England rugby players, with a mean playing exposure of 22.2 years (±5.3), completed this questionnaire study (20.3% response rate). The majority of rugby participants responded to the questionnaire as England Rugby Internationals Club (‘ERIC’) members (n = 142). Participants who were younger than 50 years (or 60 years for joint replacement) were not eligible for age-standardised SMR analyses (Fig. 1).

### Demographics

Rugby participants were aged (median (range)) 62.0 (24.2–95.0) years, whereas ELSA participants were 64.0 (50.0–99.0) years, and HSE participants 52.0 (24.0–90.0) years. The majority of participants identified as white. The mean BMI of all cohorts was > 25 kgm^2^ (28.1kgm^2^, 24.5 kgm^2^ and 27.0kgm^2^ for rugby, ELSA and HSE participants, respectively). Smoking, marital and employment status are summarised below (Table 1). Rugby participants were predominantly amateur players (83.6%) whose average total years of match play was over two decades (22.2 y).

**Table 1:** Descriptive characteristics of the rugby and ELSA cohorts. The results are expressed as mean (±SD) or number (%).

Characteristic	Rugby (N = 259)		ELSA (N = 5186)		p-value	HSE (N = 2981)		P-value
Age (yr)								
Mean (SD)	259	60.1 ± 16.1	5186	64.9 ± 10.0	<0.01	2981	53.4 ± 16.3	<0.01
Median (range)	259	62.0 (24.2–95.0)	5186	64.0 (50.0–99.0)		2981	52.0 (24.0–90.0)	
20–29		5 (2%)		0			224 (8%)	
30–39		31 (12%)		0			460 (15%)	
40–49		37 (14%)		0			603 (20%)	
50–59		50 (19%)		1916 (37%)			553 (19%)	
60–69		44 (17%)		1611 (31%)			579 (19%)	
70–79		64 (25%)		1174 (23%)			373 (12%)	
80–89		27 (10%)		447 (9%)			171 (6%)	
90–99		1 (1%)		38 (1%)			18 (1%)	
White race – no. (%)	204	199 (98%)	5148	4973 (97%)	0.46	2978	2713 (91%)	<0.01
Height (m)	252	1.82 ± 0.07	4856	1.73 ± 0.07	<0.01	2963	1.77± 0.07	<0.01
Weight (kg)	254	94.0 ± 15.2	4739	82.2 ± 13.1	<0.01	2912	84.2 ± 14.7	<0.01
BMI (kg/m^2^)*	249	28.1 ± 3.7	4720	27.5 ± 3.8	<0.02	2896	27.0 ± 4.3	<0.01
Underweight (<18.5)		0					25 (1%)	
Normal range (18.2–24.9)		41 (17%)					989 (34%)	
Pre-obesity (25.0–29.9)		148 (59%)					1272 (44%)	
Obese Class I (30.0–34.9)		50 (20%)					467 (16%)	
Obese Class II (35.0–39.9)		8 (3%)					114 (4%)	
Obese Class III (>40.0)		2 (1%)					29 (1%)	
Ever smoker	257	25 (10%)	5104	3824 (74%)	<0.01	2981	1832 (61%)	<0.01
Marital status:	207		5185		<0.01	2979		<0.01
Single/never married		6 (3%)		324 (6%)			417 (14%)	
Married		171 (83%)		3916 (76%)			1814 (61%)	
Separated		1 (1%)		64 (1%)			56 (2%)	
Divorced		14 (7%)		399 (8%)			200 (7%)	
Widowed		12 (6%)		482 (9%)			136 (4%)	
Other		3 (2%)		0			356 (12%)	
Amateur status	256	214 (84%)						
Total years of play								
Mean (SD)	245	22.2 ± 5.3						
Median (range)	245	22.0 (10.0–43.0)						
Employed or retired^1^	251	246 (98.0%)						

*Derived from self-reported height and weight: BMI = (weight in kilograms/(height in meters × height in meters)). ^1^Derived from ‘Are you currently employed’? and ‘If no, are you retired’?

### Prevalence of morbidity

The prevalence of morbidity differed between the complete cohort of rugby participants (n = 259) and ELSA participants aged 50 and above (Table 2). The most prevalent morbidities amongst rugby participants were osteoarthritis (60%), high blood pressure (28%) and joint replacement (24%), whereas the most prevalent morbidities amongst ELSA participants were high blood pressure (37%), heart problems (24%) and osteoarthritis (15%).

**Table 2:** The prevalence of morbidity in rugby players and ELSA participants

Morbidity	Rugby players (n = 259) vAged 24–95		Rugby players (n = 186) Aged 50–95		ELSA (n = 5186) Aged 50–99	
	Number of respondents	Number (%)	Number of respondents	Number (%)	Number of respondents	Number (%)
Asthma	245	24 (10%)	176	11 (6%)	5184	524 (10%)
Diabetes	238	5 (2%)	171	5 (3%)	5184	464 (9%)
High blood pressure	245	68 (28%)	178	66 (36%)	5184	1905 (37%)
Heart problems	241	43 (18%)	174	40 (22%)	5184	1236 (24%)
Stroke	198	6 (3%)	148	6 (3%)	5184	264 (5%)
Anxiety	242	18 (7%)	174	11 (6%)	5184	175 (3%)
Depression	241	15 (6%)	174	9 (5%)	5184	218 (4%)
Dementia	197	2 (1%)	147	2 (1%)	5184	36 (1%)
Osteoporosis	240*	9 (4%)	174	8 (4%)	5184	76 (1%)
Osteoarthritis	253	152 (60%)	184	112 (60%)	5017	729 (15%)
Joint replacement	253	60 (24%)	184	59 (32%)	3181^●^	202 (6%)
Hip replacement	254	39 (15%)	185	39 (21%)	3181^●^	126 (4%)
Knee replacement	254	23 (9%)	185	22 (12%)	3181^●^	80 (3%)

*denotes participant(s) ‘don’t know’ response recoded as missing. ^●^60+ for joint replacement variables.

### Health-related quality of life

Full results for the EQ-5D amongst rugby participants and HSE participants are shown in Supplementary Table S3. The highest numbers of problems were reported for pain/discomfort (78.3% and 32.6%, for rugby participants and HSE participants respectively), followed by mobility (47.4% and 18.5%, respectively). When EQ-5D domains were dichotomised to problems (2+) or no problems (1) per domain, for either the EQ-5D-5L or EQ-5D-3L, reported problems were seen to be twice as prevalent for rugby participants in comparison with the HSE reference population, within the domains of mobility, pain/discomfort and usual activities (Fig. 2). Chi-squared analyses demonstrated significant between-group differences within the domains of mobility (p = < 0.001), self-care (p = 0.041), usual activities (p = < 0.001) and pain/discomfort (p = < 0.001).

### Standardised morbidity ratios

Musculoskeletal morbidities of osteoarthritis (4.00, 95% CI 3.32 to 4.81), osteoporosis (2.69, 95% CI 1.35 to 5.38), hip replacement (6.42, 95% CI 4.69 to 8.79) and knee replacement (5.64, 95% CI 3.72–8.57) were significantly increased amongst rugby participants (Table 3). Anxiety was also seen to be more likely amongst rugby participants (2.00, 95% CI 1.11 to 3.61). Diabetes was significantly reduced amongst rugby participants (0.28, 95% CI 0.11 to 0.66). Asthma, high blood pressure, heart problems and stroke were reduced (SMR < 1), but did not reach statistical significance.

**Table 3:** Age-matched standardised morbidity ratios for rugby players aged 50+ (n = 186) against an ELSA reference population (n = 5186).

Morbidity	Prevalence	Expected prevalence*	SMR	95% CI
Asthma	11 (6%)	18 (10%)	0.60	0.33 to 1.08
Diabetes	5 (3%)	18 (10%)	**0.28**	**0.11** to **0.66**
High blood pressure	66 (36%)	71 (38%)	0.92	0.73 to 1.18
Heart problems	48 (22%)	50 (27%)	0.80	0.59 to 1.09
Stroke	6 (3%)	11 (6%)	0.52	0.23 to 1.16
Anxiety	11 (6%)	6 (3%)	**2.00**	**1.11** to **3.61**
Depression	9 (5%)	7 (4%)	1.34	0.70 to 2.58
Dementia	2 (1%)	1 (1%)	1.40	0.35 to 5.61
Osteoporosis	8 (4%)	3 (2%)	**2.69**	**1.35** to **5.38**
Osteoarthritis	112 (60%)	28 (15%)	**4.00**	**3.32** to **4.81**
Joint replacement^●^	59 (32%)	10 (5%)	**6.02**	**4.66** to **7.77**
Hip replacement^●^	39 (21%)	6 (3%)	**6.42**	**4.69** to **8.79**
Knee replacement^●^	22 (12%)	4 (2%)	**5.64**	**3.72** to **8.57**

*Denotes n to the nearest whole number. ^●^Based on HSE members 60+.

### Sensitivity Analyses

As matching to ELSA involved selecting an older subgroup of the recruited former playing population, logistic regression was used to examine the differences between the entire cohort (n = 259) of rugby participants (aged 24 to 95) and the ELSA population (aged 50 to 99). This analysis was then adjusted for age. In the entire rugby cohort (n = 259), diabetes, high blood pressure and heart problems were significantly less prevalent amongst rugby participants than ELSA participants aged 50+, in unadjusted analyses. When adjusted for age, this significant difference remained for diabetes and high blood pressure. Joint replacement, hip and knee replacement, osteoarthritis, osteoporosis and anxiety were significantly more prevalent before and after adjustment for age (Table 4).

**Table 4:** Between-cohort analyses of morbidity using the complete playing cohort (n = 259).

Morbidity	All rugby players against ELSA sample	
	Unadjusted logistic regression	Logistic regression adjusted for age
	Odds ratio (CI)	Odds ratio (CI)
Asthma	0.97 (0.63 to 1.49)	0.91 (0.59 to 1.41)
Diabetes	**0.22 (0.09** to **0.53)**	**0.24 (0.10** to **0.58)**
High blood pressure	**0.66 (0.50** to **0.88)**	**0.72 (0.54** to **0.96)**
Heart problems	**0.70 (0.50** to **0.97)**	0.77 (0.55 to 1.10)
Stroke	0.58 (0.26 to 1.32)	0.60 (0.26 to 1.38)
Anxiety	**2.30 (1.39** to **3.80)**	**1.83 (1.08** to **3.09)**
Depression	1.51 (0.88 to 2.60)	1.11 (0.63 to 1.96)
Dementia	1.47 (0.35 to 6.14)	1.56 (0.37 to 6.53)
Osteoporosis	**2.91 (1.49** to **5.70)**	**3.22 (1.64** to **6.33)**
Osteoarthritis	**8.85 (6.80** to **11.52)**	**10.12 (7.71** to **13.29)**
Joint replacement	**4.58 (3.32** to **6.33)**	**7.79 (5.47** to **11.11)**
Hip replacement	**4.40 (2.99** to **6.46)**	**6.86 (4.56** to **10.33)**
Knee replacement	**3.86 (2.38** to **6.25)**	**5.77 (3.49** to **9.52)**

### Player sentiment

Rugby participants were asked whether considering the risks and benefits of their previous participation in rugby, they would do the same again, and 94% of rugby participants either agreed, or strongly agreed. Rugby participants were also asked whether considering the benefits and risks of their previous participation in rugby, would they recommend the sport to their friends and family, and 78% agreed or strongly agreed (Table 5).

**Table 5:** Player reflections on their playing career given their experience for a subset of participants.

	1. Strongly agree	2. Agree	3. Undecided	4. Disagree	5. Strongly disagree
Considering the benefits and risks of my previous participation in rugby, I would do the same again. (n = 204)	167 (82%)	26 (13%)	9 (4%)	1 (1%)	1 (1%)
Considering the benefits and risks of my previous participation in rugby, I would recommend this to my children, relatives or close friends. (n = 204)	91 (45%)	67 (33%)	29 (14%)	13 (6%)	4 (2%)
	**1. Dramatically**	**2. Somewhat**	**3. Undecided**	**4. Not really**	**5. Not at all**
Did your rugby career enrich your life? (n = 206)	178 (86%)	27 (13%)	0	1 (1%)	0

## Discussion

### Main findings

Our study, based on data from over 250 former elite rugby players, found differences in morbidity and health-related quality of life between rugby participants and ELSA participants. Amongst participants aged 50 and above, age-matched standardised morbidity ratios show diabetes to be significantly lower amongst rugby participants (0.28, 95% CI 0.11–0.66), whereas osteoporosis (2.69, 95% CI 1.35–5.38), osteoarthritis (4.00, 95% CI 3.32–4.81), joint replacement (6.02, 95% CI 4.66–7.77), and site-specific joint replacement at the hip (6.42, 95% CI 4.69–8.79) and knee (5.64, 95% CI 3.72–8.57) are significantly more likely amongst rugby participants than ELSA participants.

Problems with health-related quality of life were most highly reported for pain/discomfort (73.8%) and mobility (47.4%). For the domains of mobility, pain/discomfort and usual activities, reported problems were twice as prevalent in rugby participants.

Sensitivity analyses using logistic regression demonstrated decreased morbidity for diabetes, high blood pressure and heart problems amongst the complete (any age) rugby and ELSA samples. When adjusted for age, a significant difference remained for diabetes and high blood pressure. Logistic regression analyses between the complete all age rugby and ELSA samples also demonstrated higher odds of joint replacement, osteoarthritis, osteoporosis and anxiety amongst rugby participants. When adjusted for age, this significant difference remained for all morbidities except anxiety.

### Strengths and limitations

Strengths of this study include the English International playing cohort and comparator population surveys being nationally representative, the consistency between self-reported outcomes across all cohorts and the comparability of question phraseology **(**see Supplementary Table S1). The cross-sectional methodology has allowed the measurement of multiple morbidities, in order to present a relatively comprehensive overview of health within this population. Limitations of this approach include reduced capacity to infer causality in estimated associations, and self-reported outcomes, as less robust measures of morbidity than direct measurement. However, this was consistent between ELSA and rugby data collection.

Morbidities may be diagnosed at any age, and our cross-sectional approach limits discussion of current morbidity, compared with lifetime morbidity. The prevalence of morbidity outcomes may also change over time in any population, and be influenced by current societal, economic and environmental factors, which we are unable to account for in this analysis. The English Longitudinal Study of Aging is one of few studies that examines the prevalence of multiple physician-diagnosed morbidities required for this comparison, however this meant limiting SMR analyses to rugby participants aged 50 and above. The sensitivity analysis using all subjects, to allow the inclusion of morbidities amongst younger rugby participants, confirmed the findings. Many of the assessed morbidities will be highly associated with age, and therefore a reference population with detailed morbidity data but a more similar age range and distribution would have been favourable. Comorbidities are also increasingly common in developed countries^32^, and this study has not explored comorbidity, interactions between morbidities, or between morbidity and health related quality of life, due to a lack of statistical power.

The elite population selected, as University attendees and International sports participants, may be of a higher socioeconomic status than the majority of the rugby playing population, and socioeconomic status is known to be positively associated with health^33^. However, these populations were selected due to their strong alumni database, which permitted us to accurately quantify response rate, and hence prevalence data. The response rate for this study was 20.3%, with a substantially higher response from ERIC members (response rate 40%), than from Oxbridge players. This response rate is lower than several population-based longitudinal studies^34,35^, and more aligned with harder to reach groups. Due to ethical limitations, we were unable to determine differences in age or socioeconomic status between respondents and non-respondents, to determine how representative respondents were of the entire cohorts. This may have resulted in a differential bias, should players who have responded be those who are older members of the lists, with a higher prevalence of morbidity than the whole target population available to participate on the lists. However, as a study of multiple general health outcomes, this should not differentially affect our overall morbidity profile for players compared with population-based survey participants, as whilst it is possible people with more illness may respond, this should affect all morbidities equally. Whilst the study presents initial findings of morbidity within this population, the results should be considered with the potential for a higher response from less healthy rugby-playing individuals. Study findings were confirmed in each cohort before this data was merged, however, and findings within the ERIC group, with a higher response rate, were similar to those of Oxbridge participants.

Morbidity and health-related quality of life amongst former elite participants may not be generalisable to the entire playing population. The majority of players do not play at the elite, particularly representative level, and these results may not represent morbidity amongst other playing populations, such as recreational players. This study was undertaken to identify initial health differences, and further study will need to determine the relevance of morbidity in elite participants, to that of recreational; and amateur players, with lower lifetime playing exposures. Female participants have been excluded from analyses due to the small number of female participants, who will have different sex-determined inherent risks of morbidity. This sample of rugby participants is also from the amateur era of rugby, and modern professional rugby is widely accepted to differ in terms of the nature and frequency of game events, its duration (ball in play time) and player physique^11^.

For rarer morbidities (<5% prevalence) in this relatively small sample, drawing robust inferences is challenging. Morbidities such as osteoporosis and dementia with low cases and wider confidence intervals will require further study in order to validate and support these findings. However, these results indicate the morbidities that most strongly differ between rugby participants and the general population, following participation in elite rugby. Future study should seek to understand the extent to which these differences in morbidity are sustained for recreational players, and in the modern era of the sport.

Parallel phraseology between comparator groups and players is considered to be a strength of this study. However, even very minor alterations in phraseology will affect morbidity sensitivity and specificity. The definition of diabetes in ELSA involved ‘diabetes or high blood pressure’ and high blood pressure in ELSA was ‘high blood pressure or hypertension’. These broader and more inclusive definitions will have increased the prevalence of these conditions amongst ELSA participants in comparison with rugby participants, and may therefore have inflated SMR results in favour of worse health in the comparator group. This may have influenced our results and should be considered when interpreting diabetes and hypertension results, as we are unable to further quantify this potential bias. A further consideration for the interpretation of results between cohorts is the difference observed between demographics for rugby participants and the general population samples. Differences particularly in age, smoking status and BMI, may affect the prevalence of morbidities, and therefore health status. Analyses were adjusted for age and weight, however not for smoking status, which may have particularly affected cardiac morbidities. However this study was designed to assess overall health status within former elite rugby players, and cannot feasibly control for all potential confounder for all morbidities, and lifestyle factors are acknowledged to be different between former elite rugby players and the general population.

### Osteoarthritis

Physician-diagnosed OA was four times higher and any joint replacement was six times higher for former elite rugby participants compared with the ELSA population. Rugby participants were asked, “Have you ever been told you have wear and tear, degeneration or Osteoarthritis by a doctor?” and including a broader definition of ‘wear and tear’ amongst this sporting population will have increased reporting of this outcome. However, ‘wear and tear’ terminology has been traditionally associated with OA^36^. OA has been established as a potential outcome following sporting injuries^17,18,37^, and this study presents initial evidence of a significant burden of lower limb OA in this population.

The extent of this difference may support proactively managing OA risk in former elite playing populations. Initial OA management includes guidance on OA care, education on the condition and non-pharmacological management with exercise and weight loss^38^. Weight management and exercise are components of a healthy lifestyle, and the emphasis of this to players transitioning from the game, and physicians in general practice, may increase awareness and begin to manage this increased 4- to 6-fold increased risk of OA outcomes.

### Cardiovascular

Cardiovascular morbidity differed between sensitivity analyses of the complete (any age) cohort and SMR analyses matched on age. In the complete cohort, heart problems and high blood pressure were significantly reduced in univariable analyses, and high blood pressure remained significantly reduced in regression analyses when adjusted for age, however no cardiovascular morbidities were reduced in SMR analyses. These differing results may reflect the increased power afforded by the complete cohort analysis, as suggested by similar SMRs, but we cannot exclude that unmatched younger rugby participants may have biased the association. The findings are consistent with previous literature showing that regular physical activity reduces the risk of cardiovascular disease and hypertension^39–43^.

### Diabetes

Diabetes is significantly reduced in former elite rugby participants. Diabetes is associated with many chronic health conditions, and predisposes individuals to cardiovascular disease, blindness, kidney disease and depression^44^. Risk factors for diabetes include age, race, family history of the condition, smoking, obesity and physical inactivity^45^. Whilst the rugby participants are younger than the ELSA sample, the standardised morbidity ratio was matched on age and as such, age could not explain this reduction. The majority of rugby and ELSA participants were white (96.6%), and therefore racial differences should also not explain the association. Although Type 2 diabetes is strongly associated with adiposity^46^, similar levels of obesity were seen between rugby participants and ELSA participants in this study. However, adiposity is assessed using BMI, which may not be a good measure of obesity in sportspersons as it may reflect high lean muscle mass rather that high fat mass: using World Health Organisation BMI categorisation, 65% of players within the semi-finalist teams of the 2003 Rugby World Cup were classified as overweight, and 25% as obese^47^. As there were no direct measures of adiposity collected for this population, it is plausible that a comparable BMI between rugby participants and the general population may disguise differences in somatotype and body composition, which may be associated with diabetes risk.

Sporting and non-sporting physical activity has been associated with reduced type 2 diabetes prevalence^48^, and, moderate intensity physical activity has been associated with approximately 30% reduction in diabetes risk (RR 0.69), sustained as a 17% reduction in risk after adjustment for BMI^49^. A higher lifetime physical activity is most likely the main contributor to the decreased risk of diabetes observed amongst rugby participants. Unfortunately, this cannot be confirmed as data on current activity was not available in both cohorts.

### Osteoporosis

The elevated SMR for osteoporosis needs further investigation. Previous studies have found higher total body bone mineral density and regional bone mineral density amongst rugby players, and physical activity has been shown to be preventative of osteoporosis^50–52^, and therefore a lower bone mineral density and osteoporosis in later life is counterintuitive. As former elite sport participants, players may have increased access to privatised healthcare, and therefore increased access to routine investigation such as Dual-energy X-ray absorptiometry (DXA) which may, through surveillance bias, increase osteoporosis diagnoses. It is also worth noting that osteoporosis was the only outcome across which several participants had declared an uncertainty in reporting. Any ‘don’t know’ responses were recoded as missing in analyses, and therefore will have not influenced this analysis directly, however may represent uncertainty around the term or recall of the diagnosis of osteoporosis.

### Anxiety

Anxiety was found to be twice as prevalent amongst rugby participants as ELSA participants, however 85% of participants reported no problems with anxiety or depression, measured using the EQ-5D. This difference may be due to the different recall period between the EQ-5D and medical history. The focus of the EQ-5D is on current health, stating for example ‘I am not anxious or depressed’ or ‘I am moderately anxious or depressed’. The difference between anxiety results may demonstrate that whilst rugby participants have previously been diagnosed with anxiety, and the rugby participants have a higher likelihood of physician-diagnosed anxiety than the reference population, this is a past diagnosis and rugby participants are not currently experiencing anxiety. All morbidities assessed were lifetime prevalence (‘ever’) physician-diagnosed morbidity and as such, may not be current.

In sensitivity analyses, anxiety was seen to be more likely before being adjusted for age in the complete cohort, and demonstrated a higher prevalence in the whole cohort as opposed to those aged 50 and above included in SMR analyses. This may suggest that anxiety is either affecting or being diagnosed more frequently amongst younger rugby participants. Potential explanations for this higher likelihood of anxiety may be character traits within elite athletes contributing to poorer mental health post-retirement, or distressing effects of career transition seen following sports participation at the elite level^53,54^, affecting mental health. Former elite rugby participants in this study will have transitioned from elite sport, which may have financial and psychological affects on players. Retirement amongst the sporting cohort may have been at an earlier age than for ELSA participants, which may affect both financial and health status, and have influenced anxiety at this time. Anxiety related mental health problems amongst University students have been previously discussed as an area of concern; more than depression and stress^55^. Therefore the University alumni in this study may be more predisposed to having a previous diagnosis of anxiety, during their life course.

### Other morbidities

Morbidities that did not significantly differ between cohorts in SMR or sensitivity analyses were asthma, stroke, depression and dementia. Stroke, dementia and depression were both rare outcomes and given the sample size, there may not be sufficient cases in each group to detect differences.

There is the potential for these findings to be translated into increased provision of population-wide targeted player welfare for former elite rugby players. Participants have demonstrated some health deficits and some health advantages when compared to an age-matched representative general population sample, and strategies such as OA-management and advice may be feasible to implement and help improve health status for retiring elite players, and players in the future.

## Conclusions

This study has substantially expanded current knowledge in long-term health and players of rugby union. Future research needs to examine the application of these findings to modern rugby, female participants and lower levels of participation and sporting exposure. Potentially modifiable risk factors that may be associated with the development of negative health outcomes need to be identified in this population.

The magnitude of musculoskeletal morbidity in this population warrants proactive education and management within this at-risk sporting population. Further research in other sports may encourage the adoption of a more proactive approach to long-term health within elite and recreational sports, encouraging healthy sporting activity for all participants.

## Electronic supplementary material

Supplementary Table S1. Phrasing and derivation of morbidity variables for ELSA and rugby cohorts.
