# Supplementary material for: Health amongst former rugby union players: A cross-sectional study of morbidity and health-related quality of life
Source: Sci Rep. 2017 Sep 28;7:11786. doi: 10.1038/s41598-017-12130-y (PMC5620077; doi:10.1038/s41598-017-12130-y)
Supplement: Supplementary file 1 — Supplementary Table S1. Phrasing and derivation of morbidity variables for ELSA and rugby cohorts. [file 41598_2017_12130_MOESM1_ESM.pdf]

Title: Health amongst former rugby union players: A cross-sectional study of morbidity and health-related quality of life

Madeleine A M Davies[1], Andrew Judge[1], Antonella Delmestri[1], Simon Kemp[2], Keith A Stokes[3], Nigel K Arden*[1], Julia L Newton*[1]

[1]Arthritis Research UK Centre for Sport, Exercise and Osteoarthritis, University of Oxford, Nuffield Department of Orthopaedics, Rheumatology and Musculoskeletal Science, Oxford, UK.

[2] Rugby Football Union, Twickenham, UK.

[3] Arthritis Research UK Centre for Sport, Exercise and Osteoarthritis, Department of Health, University of Bath, Bath, UK.

*Denotes joint senior authorship

Correspondence to: Professor Nigel K Arden,

Arthritis Research UK Centre for Sport, Exercise and Osteoarthritis, Nuffield Department of Orthopaedics, Rheumatology and Musculoskeletal Science, Botnar Research Centre, Windmill Road, Oxford, UK. OX3 7LD.

nigel.arden@ndorms.ox.ac.uk

Supplementary Table S1. Phrasing and derivation of morbidity variables for ELSA and rugby cohorts.

ELSA	Has a doctor ever told you that you have (or have had) any of the conditions on this card?	Rugby	Have you ever been told you have any of the following by a Doctor?		
Variable name	Possible response	Variable name	Phrasing	Possible response	Binary variable
hedia01-10	Value = 96.0 Label = None of these<br>Value = 1.0 Label = High blood pressure or hypertension<br>Value = 2.0 Label = Angina<br>Value = 3.0 Label = A heart attack (including myocardial infarction or coronary…<br>Value = 4.0 Label = Congestive heart failure<br>Value = 5.0 Label = A heart murmur<br>Value = 6.0 Label = An abnormal heart rhythm<br>Value = 7.0 Label = Diabetes or high blood sugar<br>Value = 8.0 Label = A stroke (cerebral vascular disease)<br>Value = -9.0 Label = Refusal<br>Value = -8.0 Label = Don't Know<br>Value = -1.0 Label = Not applicable<br>Value = 95.0 Label = Any other heart trouble (SPECIFY)	diabetes<br>high_blood_pressure<br>heart_problems<br>stroke	Diabetes<br>High blood pressure<br>Heart problems (e.g. heart attack or angina)<br>Stroke	1 Yes<br>2 No<br>3 Don't know<br>1 Yes<br>2 No<br>3 Don't know<br>1 Yes<br>2 No<br>3 Don't know<br>1 Yes<br>2 No<br>3 Don't know	Diabetes<br>Highbloodpressure<br>Heart problems<br>Stroke
hedib01-10	Value = 96.0 Label = None of these<br>Value = 1.0 Label = Chronic lung disease such as chronic bronchitis or emphysema<br>Value = 2.0 Label = Asthma<br>Value = 3.0*** Label = Arthritis (including osteoarthritis , or rheumatism)<br>Value = 4.0 Label = Osteoporosis, sometimes called thin or brittle bones<br>Value = 5.0 Label = Cancer or a malignant tumour (excluding minor skin cancers)<br>Value = 6.0 Label = Parkinson s disease<br>Value = 7.0 Label = Any emotional, nervous or psychiatric problems<br>Value = 8.0 Label = Alzheimers disease<br>Value = 9.0*** Label = Dementia, organic brain syndrome, senility or any other serious<br>Value = -9.0 Label = Refusal<br>Value = -8.0 Label = Don't Know<br>Value = -1.0 Label = Not applicable	asthma<br>dementia	Asthma<br>Dementia	1 Yes<br>2 No<br>3 Don't know<br>1 Yes<br>2 No<br>3 Don't know	Asthma<br>Dementia
heji	Have you ever had any joint replacements?<br>Value = 1.0 Label = Yes<br>Value = 2.0*** Label = No	jreplacement	Have you ever had joint replacement surgery?	1 Yes<br>2 No<br>3 Don't know	jreplacement

*** Denotes dependancy of another variable(s) on value

Supplementary Table S2. Phrasing and derivation of variables with dependencies on variables from Supplementary Table 1.

ELSA	What type of emotional,<br>nervous or psychiatric problems<br>do/did you have?	Rugby	Have you ever been told you have any of the following by a Doctor?		
hepsy1-9	Value = 1.0 Label = Hallucinations<br>Value = 2.0 Label = Anxiety<br>Value = 3.0 Label = Depression<br>Value = 4.0 Label = Emotional problems<br>Value = 5.0 Label = Schizophrenia<br>Value = 6.0 Label = Psychosis<br>Value = 7.0 Label = Mood swings<br>Value = 8.0 Label = Manic depression<br>Value = -9.0 Label = Refusal<br>Value = -8.0 Label = Don't Know<br>Value = -1.0 Label = Not applicable<br>Value = 95.0 Label = Something else	anxiety<br><br>depression	Anxiety<br><br>Depression	1 Yes<br>2 No<br>3 Don't know<br><br>1 Yes<br>2 No<br>3 Don't know	Anxiety<br><br>Depression
	Which types of arthritis do you have?		Have you ever been told you have wear and tear, degeneration or Osteoarthritis by a doctor?		
heart1-3	Value = 1.0 Label = ... osteoarthritis?<br>Value = 2.0 Label = ... rheumatoid arthritis?<br>Value = 3.0 Label = ... some other Kind of arthritis?<br>Value = -9.0 Label = Refusal<br>Value = -8.0 Label = Don't Know<br>Value = -1.0 Label = Not applicable	osteoarthritis		1 Yes<br>2 No<br>3 Don't know	Osteoarthritis
	Which joints did you have replaced?		Have you ever had joint replacement surgery? (binary variable)<br>If yes, where?		
hejia1-6	Value = 1.0 Label = Hip<br>Value = 2.0 Label = Both hips<br>Value = 3.0 Label = Knee<br>Value = 4.0 Label = Both knees<br>Value = 5.0 Label = Hip(s) and knee(s)<br>Value = 6.0 Label = Other joint<br>Value = -9.0 Label = Refusal<br>Value = -8.0 Label = Don't Know<br>Value = -1.0 Label = Not applicable	jreplacement_joint		1 jreplacement_joint__1 (Left hip)<br>2 jreplacement_joint__2 (Right hip)<br>3 replacement_joint__3 (Left knee)<br>4 jreplacement_joint__4 (Right knee)<br>4 jreplacement_joint__5 (Left ankle)<br>5 jreplacement_joint__6 (Right ankle)<br>6 jreplacement_joint__7 (Left shoulder)<br>8 jreplacement_joint__8 (Right shoulder)<br>9jreplacement_joint__9 (Left wrist)<br>10 jreplacement_joint__10 (Right wrist)<br>11 jreplacement_joint__11 (Left hand)<br>12 jreplacement_joint__12 (Right hand)<br>13 jreplacement_joint__13 (Other – please specify)	TJR_hip<br><br>TJR_knee

Supplementary Table S3. Health-related quality of life within former rugby players (EQ-5D-5L) and the Health Survey for England 2014 (EQ-5D-3L).

Dimension	Rugby players	Dimension	HSE
Mobility (n=249)		Mobility (n=2867)	
I have no problems in walking about	131 (52.6%)	I have no problems in walking about	2335 (81.4%)
I have slight problems in walking about	78 (31.3%)	I have some problems in walking about	525 (18.3%)
I have moderate problems in walking about	30 (12.1%)	I am confined to bed	7 (0.3%)
I have severe problems in walking about	10 (4.0%)		
I am unable to walk about	0		
Self-care (n=247)		Self-care (n=2856)	
I have no problems washing or dressing myself	224 (90.7%)	I have no problems with self-care	2684 (94.0%)
I have slight problems washing or dressing myself	19 (7.7%)	I have some problems washing or dressing myself	161 (5.6%)
I have moderate problems washing or dressing myself	3 (1.2%)		
I have severe problems washing or dressing myself	0		
I am unable to wash or dress myself	1 (0.4%)	I am unable to wash or dress myself	11 (0.4%)
Usual activities (n=249)		Usual activities (n=2855)	
I have no problems doing my usual activities	159 (63.9%)	I have no problems with performing my usual activities	2416 (84.6%)
I have slight problems doing my usual activities	61 (24.5%)	I have some problems with performing my usual activities	384 (13.5%)
I have moderate problems doing my usual activities	24 (9.6%)		
I have severe problems doing my usual activities	4 (1.6%)		
I am unable to do my usual activities	1 (0.4%)	I am unable to perform my usual activities	55 (1.9%)
Pain/discomfort (n=249)		Pain/discomfort (n=2859)	
I have no pain or discomfort	54 (21.7%)	I have no pain or discomfort	1925 (67.3%)
I have slight pain or discomfort	130 (52.2%)		
I have moderate pain or discomfort	56 (22.5%)	I have moderate pain or discomfort	833 (29.1%)
I have severe pain or discomfort	9 (3.6%)		
I have extreme pain or discomfort	0	I have extreme pain or discomfort	101 (3.5%)
Anxiety/depression (n=249)		Anxiety/depression (n=2857)	
I am not anxious or depressed	212 (85.14%)	I am not anxious or depressed	2384 (83.4%)
I am slightly anxious or depressed	26 (10.44%)		
I am moderately anxious or depressed	9 (3.61%)	I am moderately anxious or depressed	419 (14.7%)
I am severely anxious or depressed	2 (0.8%)		
I am extremely anxious or depressed	0	I am extremely anxious or depressed	54 (1.9%)
Health today (n=229)		Health today (n=2666)	
	79.2 (14.5)		77.7(18.0)
